# Rhizospheric bacteria: the key to sustainable heavy metal detoxification strategies

**DOI:** 10.3389/fmicb.2023.1229828

**Published:** 2023-07-24

**Authors:** Samiksha Joshi, Saurabh Gangola, Geeta Bhandari, Narendra Singh Bhandari, Deepa Nainwal, Anju Rani, Sumira Malik, Petr Slama

**Affiliations:** ^1^School of Agriculture, Graphic Era Hill University, Bhimtal, India; ^2^Department of Biosciences, Himalayan School of Bio Sciences, Swami Rama Himalayan University, Dehradun, India; ^3^Department of Life Sciences, Graphic Era (Deemed to be) University, Dehradun, Uttarakhand, India; ^4^Amity Institute of Biotechnology, Amity University Jharkhand, Ranchi, India; ^5^Guru Nanak College of Pharmaceutical Sciences, Dehradun, Uttarakhand, India; ^6^Department of Applied Sciences, Uttaranchal University, Dehradun, Uttarakhand, India; ^7^Laboratory of Animal Immunology and Biotechnology, Department of Animal Morphology, Physiology and Genetics, Faculty of AgriSciences, Mendel University in Brno, Brno, Czechia

**Keywords:** toxicity, heavy metals, detoxification, rhizospheric, bioremediation

## Abstract

The increasing rate of industrialization, anthropogenic, and geological activities have expedited the release of heavy metals (HMs) at higher concentration in environment. HM contamination resulting due to its persistent nature, injudicious use poses a potential threat by causing metal toxicities in humans and animals as well as severe damage to aquatic organisms. Bioremediation is an emerging and reliable solution for mitigation of these contaminants using rhizospheric microorganisms in an environmentally safe manner. The strategies are based on exploiting microbial metabolism and various approaches developed by plant growth promoting bacteria (PGPB) to minimize the toxicity concentration of HM at optimum levels for the environmental clean-up. Rhizospheric bacteria are employed for significant growth of plants in soil contaminated with HM. Exploitation of bacteria possessing plant-beneficial traits as well as metal detoxifying property is an economical and promising approach for bioremediation of HM. Microbial cells exhibit different mechanisms of HM resistance such as active transport, extra cellular barrier, extracellular and intracellular sequestration, and reduction of HM. Tolerance of HM in microorganisms may be chromosomal or plasmid originated. Proteins such as *MerT* and *MerA* of *mer* operon and *czcCBA*, *ArsR*, *ArsA*, *ArsD*, *ArsB*, and *ArsC* genes are responsible for metal detoxification in bacterial cell. This review gives insights about the potential of rhizospheric bacteria in HM removal from various polluted areas. In addition, it also gives deep insights about different mechanism of action expressed by microorganisms for HM detoxification. The dual-purpose use of biological agent as plant growth enhancement and remediation of HM contaminated site is the most significant future prospect of this article.

## 1. Introduction

The term “heavy metals (HMs)” represents a unique group of metals and metalloids existing naturally with high density and atomic weight. Among several HMs contamination of arsenic (As), cadmium (Cd), chromium (Cr), lead (Pb), and mercury (Hg) in environment are considered as highly toxic and are found in terrestrial, aerial, and aquatic eco-systems more than their threshold values (World Health Organization [WHO], 2010; Agency for Toxic Substances and Disease Registry [ATSDR], 2015). Commonly such metals are called as “toxic HMs” or “most problematic HMs” (Rahman and Singh, 2019). HMs are important for the growth of organisms at their optimum desirable concentrations (WHO: 0.001–3 mg/L), however at higher concentrations, these can lead to biotoxicity and have detrimental consequences on human and environmental health (Musa et al., 2013; Gupta et al., 2016). HMs are major industrial effluents, which may subsequently get accumulated in different ecosystems leading to immense threat to the various agro-ecosystems (Cheung and Gu, 2007). HM pollution has now become a crucial matter of environmental concern worldwide due to its non-biodegradability and bioaccumulation in nature (Gautam et al., 2014). Exposure of HMs exhibited severe consequences in humans (like inflammatory, respiratory, and cardiovascular diseases), animals (Engwa et al., 2019), plants (reduced growth rate, photosynthesis, and yield) (Asati et al., 2016), microorganisms (metabolism, growth, and morphology) (Ayangbenro and Babalola, 2017) and aquatic lives (death and reproduction) (Pandey and Madhuri, 2014). Worldwide, there are almost 5 million contaminated sites of soil with HMs concentration exceeding the regulatory levels (Li et al., 2019). Biogeochemical cycles on disruption leads to deposition of HM and other contaminants into aquatic and terrestrial environment like, combustion of fossil fuels, mining, nuclear power plants, industrial effluents, sludges, preservatives including organometallic compounds, dust from smelters, waste from brewery, and distillery units (Zhang et al., 2013; Gangola et al., 2023a). Apart from being toxic in nature, few HMs are also reported as essential micronutrients for efficient plant growth. HMs may also function as cofactor of several important enzymes, required for metabolism of hydrogen, involved in methane biogenesis and acetogenesis.

Metal contamination of food and water has been reported to result into several births related defects like cancer, lesion of skin, impairment of liver and kidney functions, and many more. Millions of people of Argentina, Taiwan, Bangladesh, India, Poland, China, Hungry, Japan, Belgium, North Mexico, Chile, and Mongolia are suffering from health-related issues mentioned above due to metal contamination of ground water (Tseng et al., 2005). Lead, cadmium and mercury have been given second, third and seventh rank, respectively, due to their highly toxic and widespread nature. A huge number of superfund sites were found to be HM polluted (Peters, 1999). HMs are present in different ecosystem naturally or due to anthropogenic activities like smelting of metal ores, fuel and energy production, sludge dumps, mine tailings, agricultural activities, and gas exhaust (Raskin et al., 1994). HMs impose more intensive challenge because of its recalcitrant nature and occurrence in a form that cannot undergo complete degradation but can only be complexed with some compounds or only its chemical form can be altered. Several parameters such as physical, chemical, and ecological characteristics of the polluted sites should be checked for successful achievement of bioremediation (Gu, 2021). Physicochemical and biological methods are used for remediation of sites contaminated with HMs, both of which have their own pros and cons while use of microorganisms as a biological tool is the emerging technology for degradation and remediation of pollutants at contaminated sites (Gu, 2018; Ansari et al., 2023). Microbial bioremediation is a simple, economic, ecofriendly, inexpensive, and efficient approach done for removal and detoxification of toxic pollutants using native microorganisms (Kumar Mishra, 2017; Spain et al., 2021). Removal of pollutant from any contaminated sites by employing microbial system could be achieved maximally by knowing the toxic concentration of pollutant and maintenance energy required by the microbial system (Gao and Gu, 2021). Several bioremediation methods are reported for the degradation of more than 50 pollutants, but very little innovative approach was found (Gu, 2016). Mechanisms involved mainly were biosorption, bio-oxidation and bio mineralization (Jin et al., 2018). Microbial population associated with plant roots are efficient in remediation of polluted soils and promote plant growth by several direct and indirect mechanisms such as siderophore production, phytohormone production, phosphate solubilization, biological N_2_ fixation, antibiotic production, synthesis of lytic enzymes, etc. (Goswami et al., 2016; El-Meihy et al., 2019; Joshi et al., 2023a). Exploitation of plant growth promoting bacteria (PGPB) having potential of metal detoxification as well as multiple plant growth promoting traits are promising metal bioremediation tool. Catabolic efficiency as well as production of bio surfactant and enzymes by microbes is a novel approach to enhance their remediation efficacy (Le et al., 2017). Plant growth promoting rhizobacteria (PGPR) has shown improvement in metal immobilization or mobilization in HM contaminated soils and plant biomass as they are metal resistant and phytoremediation enhancing agents (Ma et al., 2016). Removal of environmental contaminants that cause ecological imbalance is a global concern. Considering this important, this review addresses microbes as a tool for bioremediation of toxic HMs from different contaminated systems with emphasis on the involved mechanism.

## 2. Environmental presence of heavy metal

Heavy metals are present in the environment as a result of both naturally occurring pedogenetic processes and human activities such as mining, smelting, electroplating, pesticide use, release of biosolids and phosphate fertilizer (Dixit et al., 2015). HM concentrations are also influenced by weathering of minerals, erosion, volcanic activity, atmospheric deposition, and other natural sources (Fulekar et al., 2009; Sabiha-Javied et al., 2009). Unfortunately, human activity has the potential to interfere with the natural geochemical cycle of metals, causing HMs to accumulate in soil and water. When HM concentrations reach permissible levels, this can pose a risk to human health, as well as to the health of plants, animals, and aquatic life (D’amore et al., 2005). Due to excessive production from anthropogenic and natural sources, movement from mines to areas where humans are more exposed, industrial waste discharge, and increasing bioavailability, HMs end up as pollutants in soil and water. As is a metal that can be found in biosolids, insecticides, ore mining, smelting, and wood preservatives. Electroplating, phosphate fertilizers, plastic stabilizers, paints, and pigments are sources of Cd. Fly ash, steel manufacturing, and tanneries all include Cr. Biosolids, fertilizers, ore mining, pesticides, and smelting are sources of copper. Hg is present in medical waste, coal combustion, and the mining of silver. Ni can be found in surgical equipment, wastewater, culinary appliances, and automotive batteries. Pb was observed in the aerial emissions produced by the burning of used batteries, insecticides, and herbicides.

According to Lombi and Gerzabek (1998), the equation can be used to depict how HMs are distributed/mass balance in the soil’s surroundings:


$${\text{M}}_{t\,o\,t\,a\,l}=\left({\text{M}}_{p}+{\text{M}}_{a}+{\text{M}}_{f}+{\text{M}}_{a\,g}+{\text{M}}_{o\,w}+{\text{M}}_{i\,p}\right)$$

(1) $$-\left({\text{M}}_{c\,r}+{\text{M}}_{l}\right)$$

In the equation, the following variables are included: M for HM, *p* for parent material, *a* for atmospheric deposition, *f* for fertilizer source, *ag* for agrochemical source, *ow* for organic waste source, *ip* for inorganic pollutant, *cr* for crop removal, and *l* for losses due to leaching, volatilization, and other processes.

### 2.1. The bioavailability of metals present in soil

Metals in soil occur in both available and non-available forms to a microorganism (Sposito, 2000) which is directly related to positive and negatively charged salts of the metal. Various factors influencing availability of HMs in soil includes temperature, cation exchange capacity (CEC), redox potential, pH, aeration capacity, organic matter content, microbial activity in the rhizosphere, clay minerals, water quantity, root exudates, hydrous metal oxides, climate, and metal chemical properties (Roane and Pepper, 2000; Fischerová et al., 2006). Lasat (2002) showed bioavailability of HMs to be affected by some bacterial traits such as acidification, synthesis of chelating agents, and altering the redox potential. Metals exist in soluble cationic form under aerobic and oxidized conditions whereas found as insoluble carbonate or sulfides under anaerobic and reduced environment. Moreover, bioavailability of metals in free ionic form increases at acidic pH whereas reduced at high pH because of precipitation. Availability of HMs in soils is observed to be highest for zinc followed by copper, cadmium, and nickel. Metals with high CEC are observed to have reduced toxicity even at their higher concentrations (Roane and Pepper, 2000). Several reports on availability of HMs and their uptake by plants can be essential for predicting the effect of HMs on growth of plant under stressed conditions and population of rhizospheric microbes, as well as assessment of execution of bioremediation techniques for remediation of metal stressed soils. HMs are recalcitrant which affect and alters their toxicity over time. High concentration of HMs negatively affects physiology of microbes whereas also used as important micronutrient for their growth (Ahemad, 2012). Bioavailability of metals decides the possibility of interaction between metal species and bacteria that can occur either to neutralize their toxic effects or to fulfill their metabolic needs. Mostly, naturally existing microbial biomass are most effective and participate actively in detoxification mechanism, but still they are not well characterized or known. This scientific gap is due to lack of knowledge in cultivation of such unculturable microorganisms and continuous dynamic changes in their traits essential for adaptation to the existing environment. Bacteria in rhizospheric region affect HM speciation which further leads to changes in bioavailability of metals. Furthermore, they prevent phytotoxicity in plants by converting bioavailable form of HMs to their non-bioavailable forms in soils (Jing et al., 2007). Bioavailability of metal varies from species to species. Speciation of metals and the resulting bioavailability decides the overall toxicity as well as physiological effects of a metal on biological systems (Knotek-Smith et al., 2003). Nutrient status of the soil also affects the bioavailability of HMs. Several *in situ* experiments rely on the enrichment of the existing microbial population by adding nutrients such as carbon, nitrogen, phosphorus, etc., called bioaugmentation. Most of the degradation processes are oxidative in which microorganisms harness energy through electron exchange. The availability of the oxygen is the major kinetic obstacle to the aerobic microbes due to low solubility in water.

## 3. Heavy metals and ecotoxicity

According to Gao and Gu (2021) at a particular concentration of pollutant or below its toxicity limit, microorganisms require lower energy for their maintenance, isolation, enrichment, and their efficient utilization for biodegradation. HMs are categorized according to their reactivity, effect on biological systems and their target sites. Some of them have vital role (such as zinc, iron, copper, magnesium, and calcium) in biological system whereas others are also reported to have carcinogenic and cytotoxic effects (like mercury, lead, and cadmium). Rapid industrialization, urbanization as well as mining activities have altered biogeochemical cycling which further raised accumulation of HMs in terrestrial, atmosphere, and aquatic eco-system. Such changes may severely affect the biotic communities present on those sites. Various reports showed HM stress on higher plants like cadmium, lead, and mercury inhibiting chlorophyll biosynthesis, respiration as well as oxidative stress which may further cause cellular damage, disturbs ionic balance of the cell, and toxicity in higher organisms (Mithoefer et al., 2004; Han F. X. et al., 2006; Han S. H. et al., 2006). Toxic HMs contaminates groundwater and biota and have negative effects on health of human beings. It is essential to examine the distribution and concentration of toxic HMs in riverine ecosystems (Islam et al., 2018). Major sources of contamination in aquatic system include domestic sewage, industrial effluents, agricultural run-off, and mining operations (Zhuang et al., 2013). Water contamination with HMs is a sensitive environmental issue which adversely affects surface water, ground water, plants, human, animal’s health (Rezania et al., 2016), aquatic organisms (Abrar et al., 2011) as well as alters histopathological tissues of aquatic organisms (Ahmed et al., 2014). HM contaminated water bodies are a severe concern worldwide because of biomagnifications, environmental persistence, bioaccumulation, and toxicity (Rajaei et al., 2012). Contamination of riverine sediments by HMs may cause ecological risk to organisms present in benthic region (Pacle Decena et al., 2018). Soil with more inputs of fertilizers is enriched with HMs. Bioavailability of HMs varies according to physicochemical properties and metal speciation of soil which is further essential for plant uptake. Urban areas soil has been reported with high amount of lead, out of which only up to 85% is bio accessible (Mackay et al., 2013). Non-essential HMs are reported to be hazardous and highly toxic to plants, humans, and animals even at minimum concentrations (Mahboob et al., 2014). Also, few essential HMs may increase risk of toxicity if used at increased concentrations as listed in Table 1. Metals that are toxic, persistent, and bio accumulative are more harmful (DeForest et al., 2007). Several HMs have been referred as teratogenic, mutagenic, and carcinogenic. Cadmium with potential of bioaccumulation and high toxicity resulted into population decline of freshwater mussels (Ngo, 2007).

**TABLE 1:** Impact of HMs on plant growth and their toxicological effects on microbes.

Heavy metal	Distribution	Essential/Non-essential	Role in plants	Toxicity effect on plants	Toxic effect on microbes	References
Cu	Lakes, earth’s crust, rivers, and oceans	Essential	Photosynthesis; synthesis of ATP and CO_2_; essential part of some proteins like cytochrome oxidase and plastocyanin; cofactor for several enzymes like superoxide dismutase, dioxygenase and ascorbate oxidase.	Cu causes cytotoxic effects, induces stress, reduced plant growth, generates reactive oxygen species, chlorosis in leaf.	Inhibition of several enzyme activities and cellular functions.	Chatterjee et al., 2006; Adrees et al., 2015; Habiba et al., 2015; Fashola et al., 2016; Sayqal and Ahmed, 2021
Cd	Soil, sedimentary rocks, and water	Non-essential		Cd toxicity can cause reduced availability of essential elements such as iron, calcium, phosphorus, and magnesium; stunted growth, browning of roots, chlorosis; several cytotoxic effects; decrease in nitrogen fixation.	Impairment of different proteins, DNA and RNA; interference with transcription process and cell division.	Balestrasse et al., 2003; Guo et al., 2008; Asgher et al., 2015; Fashola et al., 2016; Khan et al., 2016; Sayqal and Ahmed, 2021
Zn	Surface water, soil, and rock	Essential	Plant growth and cell metabolism; gene expression, activation of enzyme, gene regulation, protein synthesis; cofactor in metabolic pathways of different biomolecules; reproductive development.	Zn toxicity may lead to inhibition of growth and several plant metabolic functions; restricts root and shoot growth, chlorosis; interfere with uptake of other important elements like copper and manganese.	Decline in biomass as well as growth.	Foy et al., 1978; Fontes and Cox, 1998; Cakmak, 2000; Malik, 2004; Dhankhar et al., 2012; Sayqal and Ahmed, 2021
As	Soil and volcanic eruption	Non-essential		As(V) is analog to PO_4_^3+^ therefore competes with uptake of PO_4_^3+^ which further have negative effects on ATP production, oxidative phosphorylation and transport system; arsenic toxicity may cause growth inhibition, low yield, free radical and ROS formation, protein activity inhibition, deficiency of other essential elements.	Inhibition of enzyme activities.	Tripathi et al., 2007; Gunes et al., 2009; Sankarammal et al., 2014; Kumar et al., 2015; Anjum et al., 2016; Sayqal and Ahmed, 2021.
Ni	Air, soil, sediments, and water	Essential	Essential part of different metalloenzymes such as urease, hydrogenases, superoxide dismutases, methyl coenzyme M reductase, RNase-A, dehydrogenases, acetyl Co-A synthase.	High concentration of nickel can cause chlorosis, necrosis, and wilting; impairment of photosynthesis, sugar transport, and water balance; negative effects on balance of nutrients and ATPase activity leading to impaired functions of cell membrane.	Negative effects on cell membrane, oxidative stress, and deactivation of various enzymes.	Nakazawa et al., 2004; Sethy and Ghosh, 2013; Fashola et al., 2016; Sayqal and Ahmed, 2021
Cr	All environments	Non-essential		Cr toxicity can result in chlorosis, growth inhibition, and low synthesis of photosynthetic pigments; low uptake of important elements like iron, phosphorus, calcium, magnesium, potassium; inhibition of ETC (electron transport chain) photophosphorylation, and some enzyme activities; disorganization of chloroplasts.	Inhibition of growth, oxygen uptake; extension of lag phase.	Cervantes et al., 2001; Peralta-Videa et al., 2009; Vikram et al., 2011; Ahemad, 2015; Sayqal and Ahmed, 2021
Pb	Soil	Non-essential		Lead accumulation may cause several deleterious direct and indirect effects on physiology, morphology and biochemical functions of plants; negative effects on membrane permeability, enzyme activities, nutrition, growth hormones water uptake, ATP synthesis, lipid peroxidation; synthesis of ROS in large amount leading to damage of DNA.	DNA and protein denaturation, transcription termination and stop enzymatic regulation.	Sethy and Ghosh, 2013; Fashola et al., 2016; Sayqal and Ahmed, 2021
Mn	Earth’s crust	Essential	As cofactor, in photosynthesis and form metalloproteins.	Mn toxicity can reduce efficiency of photosynthesis; cause necrosis, cracks in root, chlorosis, and brown coloring of leaf, stem and petiole.	Negative effects on metabolic functions and respiration.	Bachman and Miller, 1995; Foy et al., 1995; Kitao et al., 1997; Amorim et al., 2018
Hg	Water, soil, and air	Non-essential		Toxic amount can result in visible injuries and physiological issues in plants; synthesis of reactive oxygen species and inhibition of mitochondrial activity; negative effects on cellular metabolism in plants; interferes with dark and light reactions of photosynthesis.	Impairment of cell membrane, deactivation of proteins and enzymes.	Cargnelutti et al., 2006; Zhou et al., 2007; Fashola et al., 2016.

## 4. Challenges and possible solution for heavy metal detoxification

Undoubtedly, the combination of ecological analysis and chemical data can offer greater understanding of the environmental condition of ecosystems and the transformations that have occurred (Gu, 2014). HMs cannot be subjected to complete degradation, i.e., modification in the nuclear structure does not occur, thus they can only undergo change in their oxidation states which in turn changes their chemical behavior (Sobariu et al., 2017). Change in oxidation may lead to various consequences like solubility of metal increase in water that can be further easily removed by leaching or may decrease its solubility by which it becomes less available for decontamination, may get converted to less toxic forms or can be removed from polluted sites via volatilization (Garbisu and Alkorta, 2003).

The root of plants provides aeration to the soil and influences the distribution of rhizospheric microbes through soil. The root system is also capable of penetrating the impermeable zones of soils and draw the soluble forms of the organic contaminants. A significant symbiotic relationship between the plant root system and rhizospheric microbes is observed and this relationship has long been exploited for rhizo-remediation (Gangola et al., 2022a). Successful rhizo-remediation is dependent upon several factors such as: root and rhizosphere colonization by microbes, formation of various useful metabolites by the plants, survival, and ecological interactions with other organisms. This novel approach can also be called phytoremediation. The employment of leguminous plants for bioremediation has seen a rise in the recent times due to immense bioremediation ability and capability of biological nitrogen fixation as well as root nodule formation (Pastor et al., 2003; Kamaludeen and Ramasamy, 2008; Gangola et al., 2022b). Rhizospheric microbes have immense potential of nutrient cycling, restoration of soil structure, decontamination of various contaminants, pest management, and enhanced plant growth (Gangola et al., 2023b). Therefore, soil microbes can improve the bioremediation potential of plants and in turn lower the phytotoxicity of the soil contaminants. In symbiotic associations between microbes and plants, plant make carbon source available to the associated microbes which in turn helps in reduction of phytotoxicity of the soil contaminants. In addition, some non-specific associations are also observed in between microbes and plants where metabolic processes of plant stimulate the microbial growth, which in turn degrade the soil pollutants due to their inherent metabolic activity. Plants roots are capable of releasing root exudates and increasing the metal ion solubility. These biochemical processes increase the bioremediation potential of rhizospheric microbes associated with the plant roots and thus is of significance for remediation of heavy-metal pollution soils (Gangola et al., 2021, 2022c). This association also accounts for effective phytoremediation due to potential of microbes to influence bioavailability and solubility of the HM. PGPRs boost up the growth and development as well as improve the metal tolerance by decontaminating the toxic HMs (Tassi et al., 2008).

### 4.1. Bioremediation

**FIGURE 1:**
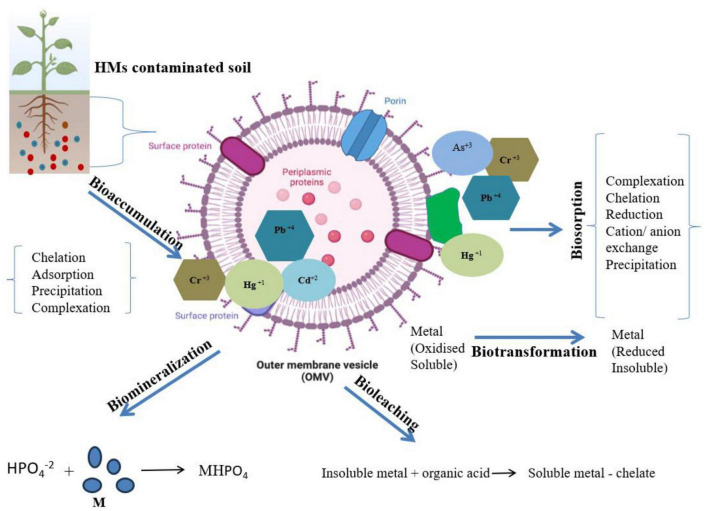
Different strategies such as: bioleaching, biomineralization, biotransformation, biosorption, and bioaccumulation by microbial system for the removal or transformation of toxic HMs from contaminated sites.

Bioremediation offers transformation, degradation, or detoxification of hazardous compounds, organic or inorganic waste by natural biological activity of bacteria, fungi, or plants in the environment (Gu and Pan, 2006; Gu and Wang, 2013; Siddiquee et al., 2015; Gu, 2021). This process is less expensive; low technology based and can be done on site as well as it can be improved by addition of nutrient, an electron acceptor, potent microbial population, pH, soil type, and temperature (Vidali, 2001; Gu, 2019). Moreover, alteration of environmental parameters to provide optimum conditions for microbial activity and growth allow bioremediation to be more effective and proceed at a faster rate (Gu, 2003; Su, 2014). Microbes with ability to remediate when applied to the contaminated site results into transformation of toxic compounds via several metabolic reactions (Siddiquee et al., 2015). Efficient biodegradation and remediation of pollutants at contaminated sites can be achieved by the knowledge of microbial biochemical reactions along with its engineering and management (Gu, 2021; Joshi et al., 2023b). Several approaches for bioremediation include bioreactor, biosorption, biostimulation, bioventing, bio filters, composting, bioaugmentation, and land farming. Several reports have demonstrated efficient elimination of HMs or their conversion to benign or less toxic forms using potent microbes (Qazilbash, 2004). Potential of microbes of exploiting available nitrogen or carbon source for their growth and survival is used in bioremediation and thus bacteria utilizes the contaminants as source of nutrient (Tang et al., 2019). Soil microbes in rhizospheric zone are considered as efficient degraders to overcome HM stress. Other than these plants also possess several detoxifying strategies like synthesis of thiols with high ability to take up HMs. MacNaughton et al. (1999) showed bioremediation as a significant technology to detoxify ground water, soils, and coastal water bodies. Interaction of microbes with HMs can occur via different mechanisms as shown in Figure 1, such as biomineralization, biotransformation, bioleaching, biosorption, etc., which further helps in metal bioremediation (Pande et al., 2022). Several investigations as listed in Table 2, revealed that bacterial communities belonging to genus *Escherichia, Bacillus*, and *Mycobacterium* isolated from various contaminated sites are found efficient for the removal of HM (Cd, Cr, and Cu) (Jiang et al., 2015). Similarly, they can be remediated by fungal genera, such as *Pleurotus, Acremonium*, and *Fusarium*. Li et al. (2019) discovered microbial consortia with high remediation potential of HMs. Composting and Immobilization are used to enhance the rate of remediation by increasing the activity of microorganisms (Poulsen and Bester, 2010; Chen et al., 2015). Rhizobium-legume symbiotic association is a significant bioremediation method for metal contaminated soil and have been exploited for removal of arsenic from soils. Mandal et al. (2011) have reported isolation of arsenate (2.8 mM arsenate) resistant symbiotic bacteria *Rhizobium* strain (VMA301) from roots of *Vigna mungo.* This resistant strain showed efficient N_2_ fixation as well as helped in decontamination of arsenic containing soil. However, a delayed nodulation and reduced nitrogenase activity was also observed in arsenic treated plants. Arsenic was found to accumulate in roots at higher rate than in the root nodules. Although a lot of research has been done on environmental pollutant degradation but still waiting for the need of efficient biodegradation technology (Gu, 2016).

**TABLE 2:** Heavy metal mediated toxicological effects and microorganisms involved in the process of removing them from soil.

Heavy metal	Toxicological effects	Microorganisms involved	Process of removing heavy metal	References
Cd, Pb	Cancer, toxicity to different biological system such as skeletal, reproductive, urinary, cardiovascular, respiratory, and nervous system	*Pseudomonas aeruginosa* BS2	Di-rhamnolipid based soil washing	Juwarkar et al., 2007
Cd, Zn, As	Stomach pain, nausea and vomiting, gastrointestinal and kidney dysfunction, nervous system disorders, cancer	*Pseudomonas aeruginosa* LFM 634	Soil washing and chemical precipitation with the help of rhamnolipid	Lopes et al., 2021
Cd, Co, Pb, Zn, Ni	Low levels of energy, dysfunctioning of brain, damage lungs, kidney, and liver, disturbed blood, dermatitis and ulcers, increases the occurrence of risk to lung, nasal, sinus, anemia, dizziness, nausea, vomiting, diarrhea, cancer	*Bacillus subtilis* A21	Soil washing with the help of lipopeptide	Singh and Cameotra, 2013
Cu, Zn	Abdominal pain, diarrhea, nausea, vomiting, blue/green colored faces, fatigue, anemia, and dizziness	*Pseudomonas aeruginosa* ATCC 9027	Soil washing in presence of rhamnolipid	Mulligan et al., 2001
As, Cu, Zn, Pb	Dysfunctioning of kidney, nervous system failure, lesions on skin, vascular damage, weakened immunity congenital disorder and cancer	*Pseudomonas aeruginosa*	Soil flushing in presence of rhamnolipid (JBR-425)	Mulligan, 2009
Pb, Ni, Cd, Ba, Zn	Nausea, vomiting, diarrhea, headache, cough, shortness of breath, anemia, cancer	*Pseudomonas aeruginosa*	Adsorption by rhamnolipid	Elouzi et al., 2012
Cu	Nausea and vomiting	*Pseudomonas aeruginosa* MTCC2297	Soil washing in presence of rhamnolipid	Venkatesh and Vedaraman, 2012
Cu, Zn	Abdominal pain, diarrhea, nausea, vomiting, blue/green colored faces, fatigue, anemia, and dizziness	*Torulopsis bombicola* ATCC 22214	Soil washing in presence of sophorolipid	Mulligan et al., 2001
Cr, Cd, Pb, Zn, Cu	Dermatitis, ulcers, lung disease risk increase, sinus, anemia, and dizziness, pulmonary sensitization, cancer	*Candida bombicola*	Sophorolipid	Ahuekwe et al., 2016
Cd, Pb	Gastrointestinal disorders, dysfunctioning of kidney, nervous system disorders, weakened immune system, congenital defects, cancer	*Starmerella bombicola* CGMCC 1576	Sophorolipid dependent soil washing	Qi et al., 2018
Zn	Abdominal pain, nausea, and vomiting	*Cryptococcus* sp. VITGBN2	Sophorolipid based immobilization	Basak and Das, 2014
As	Gastrointestinal disorders, dysfunctioning of kidney, nervous system disorders, weakened immune system, cutaneous rash congenital defects, cancer	*Candida bombicola*	Soil washing with the help of sophorolipid (SL18)	Arab and Mulligan, 2018
Zn, Cu	Stomach pain, nausea, and vomiting	*Candida tropicalis* UCP 0996	Soil washing with the help of lipopeptide	da Rocha Junior et al., 2019
Hg, Pb, Mn, Cd	Gastrointestinal disorders, dysfunctioning of kidney, nervous system disorders, weakened immune system, cutaneous rash congenital defects, cancer, damage of lungs and liver, cancer	*Bacillus* sp. MSI 54	Co-precipitation	Ravindran et al., 2020
Pb, Cd, Cr	Eczema and skin ulcers that are typically painless, lung disease risk increases, sinus, nasal, anemia, dizziness, cancer	*Bacillus cereus*	Soil washing with the help of lipopeptide	Ayangbenro and Babalola, 2020
Cu, Zn	Stomach pain, nausea, and vomiting	*Bacillus subtilis* ATCC 21332	Surfactin based soil washing	Mulligan et al., 2001
Cu, Zn, Cr, Cd	Eczema and skin ulcers that are typically painless, lung disease risk increases, sinus, nasal, anemia, dizziness, cancer	*Bacillus* sp. HIP3	Metal chelation by surfactin	Md Badrul Hisham et al., 2019
Zn, Cu	Stomach pain, nausea, and vomiting	*Candida lipolytica* UCP 0988	Precipitation-dissolution	Rufino et al., 2012
Fe, Zn	Conjunctivitis, choroiditis, and retinitis, stomach pain, nausea, and vomiting	*Candida lipolytica* UCP 0995	Soil washing	Luna et al., 2016
Cu, Cr	Abdominal pain, nausea, vomiting, irritation in the respiratory tract, pulmonary sensitization, lung disease risk increases, nasal, sinus cancer	*Rahnella* sp. RM	Soil washing	Govarthanan et al., 2017
Al, Pb, Zn, Cd, Fe, Cu, Mn	Immune system disorder, oxidative stress, mutagenic to DNA, inflammation, protein denaturation, inhibition of metabolic enzymes, apoptosis, dysplasia, increase lung risk, liver and kidney damage, cancer	*Citrobacter freundii* MG812314.1	Precipitation	Gomaa and El-Meihy, 2019
Pb, Mn, Cu, Zn, Cd, As	Low levels of energy, dysfunctioning of brain, damage lungs, kidney, and liver, disturb composition of blood, dermatitis, and ulcers, increases the occurrence of risk to lung, nasal, sinus, anemia, dizziness, nausea, vomiting, diarrhea, cancer	*Burkholderia* sp. Z-90	Combined bioleaching and flocculation	Yang et al., 2018
Pb, Cd	Dysfunctioning of brain, damage lung, kidney, and liver, change the blood composition, eczema, and skin ulcers that are typically painless cancer	*Bacillus circulans*	Co-precipitation	Das et al., 2009
Cr	Irritation in the respiratory, pulmonary sensitization, hypersensitivity	*Bacillus* sp.	Reduction	Gnanamani et al., 2010
Cd, Pb	Dysfunctioning of brain, damage lungs, kidney, and liver, can change blood composition, eczema and skin ulcers that are typically painless, cancer	*Pseudomonas* sp. LKS06	Biosorption	Huang and Liu, 2013

### 4.2. Mechanisms utilized in heavy metal bioremediation

*Biosorption* by microorganisms remediates the contaminated sites or HMs through binding with extracellular polymers or adsorption on the cell surface (Gangola et al., 2018). The outer cell shield of microbes carries active groups of compounds which provides sorption ability and further helps in binding of metals. Such linking occurs due to negative potential of active groups present on outer membrane and cationic metal ions. Biosorption is a reversible process which makes it essential for both recovery [for non-toxic and essential HMs such as gold (Au), Zinc (Zn), and copper (Cu)] and removal of HMs. Various studies showed binding of Cr^3+^, Cd^2+^, Pb^2+^, Cu^2+^, Co^2+^, Zn^2+^, and Ni^2+^ to microbial biomass by sorption at pH between 5 and 7 and get liberated at low pH whereas few metal ions like Ag^2+^ and Au^2+^ stay bound to biosorbents even at low pH (Kisielowska et al., 2010). Microorganisms are found as rapid adsorbers of HM ions for example *Bacillus* sp. showed 60% sorption of its Cu^2+^ at pH 7.2, at initial phase and reached to adsorption equilibrium within 10 min (He and Tebo, 1998). Several studies demonstrated good binding affinity of microbes via biosorption such as *Streptomyces rimosus* for iron and lead (Sahmoune, 2019), *Staphylococcus hominis* strain AMB-2 for cadmium and lead (Rahman et al., 2019), *Cronobacter muytjensii* KSCAS2 for multiple HMs (Saranya et al., 2018), and *Staphylococcus aureus* biofilms for U(IV) remediation (Shukla et al., 2020).

*Bioleaching* process mainly involves complexation, biological dissolution, bio-oxidation (Jin et al., 2018), or conversion of sparingly soluble metal compounds into easily soluble forms which can be removed easily (Kisielowska et al., 2010). Various microorganisms known as potent metal mobilizers produces low molecular weight organic acids such as tartaric acid, oxalic acid, gluconic acid, malic acid, citric acid, and butyric acid for the solubilization of HMs from the insoluble ores and exudation of complexion agents that can easily dissolve HMs from soil particles comprising HM minerals. Application of *Acidithiobacillus thiooxidans* and *Acidithiobacillus ferrooxidans* in metal leaching is proven to be effective because of their biochemical attributes as well as high resistance to temperature and pH (Błaszczyk, 2007). Few studies showed that rate of cadmium leaching promoted by providing more nutrients to microbes for increased production of organic acids (Jin et al., 2018). Furthermore, a few genera such as *Citrobacter* synthesized free inorganic phosphate with high potential to trap toxic metal ions and forming insoluble metal phosphates (Marchenko et al., 2015). Some efficient strains of bacteria like *Bacillus licheniformis* and *Corynebacterium* sp. are reported to perform oxidation-reduction reactions and convert the valance of HMs leading to alter their toxicity and mobility (Gavrilescu, 2004). Acidophiles are mainly involved in bioleaching (Srichandan et al., 2014). Arsenic removal by bioleaching using individual and mixed culture of *A. ferrooxidans* and *A. thiooxidans* was reported by Zhang and Gu (2007). *Bioprecipitation* and *biocrystallization* of HM compounds by microbes results in conversion of metal into form which is less toxic. This occurs mainly due to enzymatic activities (Sklodowska, 2000). Transformation of toxic metal by microbes may occur by several reactions like demethylation, methylation, oxidation, and reduction. For example, Gram-positive bacteria obtained from tannery sewers, transformed highly toxic chromium (VI) to its less toxic form chromium (III) (Kisielowska et al., 2010). *Bioaccumulation* is a toxic kinetic process in which active metabolism leads to HM uptake of contaminants inside the organism when the rate of absorption of contaminant exceeds its rate of loss (Chojnacka, 2010). Microbes with bioaccumulation potential should have high tolerance to variety of contaminants as well as, superior bio transformational ability to make it non-toxic in nature (Mishra and Malik, 2013). This is not considered as much valuable approach as after certain amount of accumulation it can exert toxic effects to cells. Studies showed removal of HM to be more effective by bioaccumulation as compared to biosorption (Henriques et al., 2015). In an investigation, biomasses of *Bacillus megaterium* and *Bacillus circulans* demonstrated efficient removal of toxic form of chromium (VI) at much high rate (32–34.5 mg g^–1^ dw) by bioaccumulation as compared to biosorption (15.7–39.9 mg g^–1^ dw) (Srinath et al., 2002). Bioaccumulation potential was shown by *Pseudomonas putida* 62 BN for cadmium and *Bacillus cereus* M116 for nickel (II) (Rani and Goel, 2009; Naskar et al., 2020). Microbial transformation results in reduced toxicity and vitalization of HMs. Some reports showed detoxification of arsenic (III) by *Acinetobacter* sp. as well as *Micrococcus* sp. and chromium (VI) by *Bacillus* sp. SFC 500-1E (Tayang and Songachan, 2021). Some of the key bacterial HMs resistant mechanisms are explained.

#### 4.2.1. Extracellular barrier

The cell wall, plasma membrane, and capsule act as an extracellular barrier to stop metal ions from entering the cell (El-Helow et al., 2000). Through ionizable groups like amino, carboxyl, hydroxyl and phosphate on their cell wall or capsule, bacteria can bind metal ions. According to Pardo et al. (2003), this adsorption is a passive process that can even take place in dead bacterial cells. The two phases involved in the accumulation of metal ions by living cells are gradual active transport into the cytoplasm and non-specific initial adsorption by the cell wall (McEldowney, 2000).

*Acinetobacter* sp. (Irawati et al., 2015), *Marinobacter* sp. (Bhaskar and Bhosle, 2006), *Klebsiella* sp. (Mohan et al., 2019), and *Enterobacter cloacae* (Iyer et al., 2005) are just a few of the bacteria whose capsules have been shown to accumulate metal ions primarily through carboxyl groups of their polysaccharides. Exopolysaccharide (EPS) production by copper-tolerant *Pseudomonas aeruginosa* strains is twice as high as that of copper-sensitive strains (Kazy et al., 2002). Metal ions, however, can also stop bacteria from producing EPS. Multiple copper-tolerant but deficient in the synthesis of EPS gellan *Sphingomonas paucimobilis* mutants were found by Richau et al. (1997). The authors hypothesized that because EPS synthesis is a very energy-intensive process, the mutants’ higher copper tolerance was caused by a slower growth rate and the use of stored energy for defense against metal stress.

#### 4.2.2. Active transport

The majority of HM resistance methods used by bacteria, sometimes referred to as efflux, export metal ions from cells. On chromosomes (Franke et al., 2001; Lee et al., 2007) and plasmids (Nies, 2000), efflux system genetic factors can be discovered. The magnesium transport system allows the entry of cobalt, cadmium, nickel, zinc, and manganese ions to the *Ralstonia metallidurans*, whereas some metal ions can enter the cell through the essential element uptake systems such as chromate which is transported via the sulfate transport system (Gonzalez Henao and Ghneim-Herrera, 2021). Through ATP hydrolysis or an electrochemical gradient (Mathivanan et al., 2021), metal ions are exported from the cell. Proteins from three families make up efflux systems: P-type ATPases, CDF (cation diffusion facilitator), and RND (resistance, nodulation, cell division). Gram-negative bacteria’s P-type ATPases and CDF proteins move particular substrates across the plasma membrane and into the periplasm. It is important to note that CDF proteins specifically interact with divalent metal ions (Zn^2+^, Co^2+^, Ni^2+^, Cd^2+^, and Fe^2+^), whereas P-type ATPases primarily transfer metal ions that have a high affinity for sulfhydryl groups (Cu^+^/Ag^+^, Zn^2+^/Cd^2+^/Pb^2+^). RND proteins assemble into transport complexes that carry cations from the periplasm across the plasma membrane (Nies, 2003).

The efflux system that allows the multi-resistant bacterium *R. metallidurans* CH34 to tolerate copper, cobalt, and zinc ions is encoded by the Czc operon. The system makes use of an electrochemical gradient and is made up of the *CzcCB2A* efflux complex, which also functions as a cation-proton antiporter and includes the subunits *CzcC, CzcB*, and RND-protein *CzcA*. While *CzcA* might contribute to some degree of HM resistance, *CzcC* and *CzcB* are necessary for the efflux system to operate properly (Nies, 2000). With CPx-type ATPases discovered in bacteria like *Enterococcus hirae* (*CopA* and *CopB*), *Streptococcus mutans* (Vats and Lee, 2001), and *Escherichia coli* (Rensing et al., 1999), the P-type ATPase family comprises transporters for mono- and divalent metal cations. In bacteria like *Staphylococcus aureus* and *P. putida*, P-type ATPases called *CadA* and *ZntA* play a role in cadmium tolerance (Oger et al., 2003; Lee et al., 2007).

Certain bacteria can use other mechanisms in addition to efflux systems to resist HMs (Saxena et al., 2002). For instance, the *P. putida* strain S4 transports copper ions from the cytoplasm and sequesters them in the periplasm using an ATPase efflux mechanism. Another illustration is the *ars* system, which has 3–5 genes and is found in both Gram-positive and Gram-negative bacteria. The *ArsA/ArsB* ATPase pump and the *ArsC* reductase are both encoded by the *ars* operon. According to Mukhopadhyay et al. (2002), in the first stage, cytoplasmic *ArsC* arsenate reductase enzymatically converts arsenate to arsenite, which is then exported by the efflux system through the plasma membrane.

#### 4.2.3. Intracellular sequestration

Through a procedure known as intracellular sequestration, diverse substances in cell cytoplasm combine metal ions. Two kinds of eukaryotic metal-binding peptides—metallothioneins and phytochelatins—are high in cysteine residues and bind metal ions via sulfhydryl groups (Pinto et al., 2003). For metallothionein synthesis, which is triggered by cadmium and zinc ions, *Synechococcus* sp. encodes two genes, *smtA* and *smtB* (Ybarra and Webb, 1999). Low-molecular-weight proteins with a high cysteine content enable a cadmium-tolerant strain of *P. putida* to sequester copper, cadmium, and zinc ions intracellularly. According to Ivanova et al. (2002), some marine gamma-proteobacteria synthesize phytochelatin-like low-molecular-weight proteins that are cadmium-inducible. Glutathione is used by *Rhizobium leguminosarum* cells to sequester cadmium ions intracellularly (Lima et al., 2006).

#### 4.2.4. Extracellular sequestration

Metal ion accumulation in the periplasm or outer membrane of microbial cell, or their fusion as insoluble compounds, is referred to as extracellular sequestration. For instance, copper-resistant strains of *Pseudomonas syringae* produce *CopA, CopB*, and *CopC*, which bind copper ions and cause copper to build up in the periplasm or outer membrane, resulting in blue bacterial colonies (Cha and Cooksey, 1991). Similar to how copper-tolerant *Pseudomonas pickettii* US321 builds up copper ions in the periplasm or outer membrane, resistant strains likely store copper as a complex and transport it into the cytoplasm, while sensitive strains likely build up copper in a toxic free ionic form that damages cells (Gilotra and Srivastava, 1997). *Pseudomonas stutzeri* AG259, which was isolated from the soil of a silver mine, accumulates silver ions as sulfide complexes on the cell surface or in elemental form in the periplasm due to plasmid-encoded resistance to high concentrations of silver ions in the medium (Haefeli et al., 1984; Slawson et al., 1992; Klaus et al., 1999). Some bacteria release metal ions from the cytoplasm into the periplasm, where they are then trapped. The periplasmic protein *SilE* of the *Salmonella* sp. strain selectively binds silver ions, which are then exported by the ATPase pumps *SilCBA* and *SilP* (Silver, 2003).

#### 4.2.5. Reduction of HM ions

Smirnova (2005) claims that a variety of HM ions, such as chromate, molybdate, and vanadate, are reduced by bacteria from various biological niches. Some bacteria can use metals and metalloids as electron acceptors or donors to produce energy. During anaerobic respiration, bacteria can use oxidized metals as terminal electron acceptors, and enzymatic reduction of metal ions can result in the creation of less hazardous forms of HMs including mercury and chromium (Barkay et al., 2003; Viti et al., 2003).

The *mer*-operon, which imparts tolerance to mercury by encoding proteins like *MerT* and *MerA*, is one of the best-studied mechanisms for metal detoxification (Brown et al., 2002). Divalent mercury ions can enter the cell by the *MerT* transport protein and are then converted to elemental mercury by the intracellular *MerA* reductase.

According to Nies (2003), some plasmids or transposons that can be shared by different bacteria through horizontal gene transfer (HGT) contain genetic determinants of HM tolerance. Natural transformations occurring frequently, the isolation of plasmid- and transposons-bearing bacteria from a variety of environments, and the acquisition of new traits by autochthonous microbiota after exposure to plasmid-bearing bacteria are all evidence supporting the role of HGT in bacterial evolution under changing environmental conditions (Coombs and Barkay, 2005).

Bacterial plasmids were where metal resistance mechanisms were first discovered (Summers and Silver, 1972). The *Ars* operons on the chromosomes of *E. coli*, *P. aeruginosa*, and *Bacillus subtilis*, which structurally mimic plasmid-borne genetic determinants, are examples of metal resistance systems that are similar to those on plasmids (Mukhopadhyay et al., 2002). However, hazardous metal resistance genes are carried on plasmids while important metal ion homeostasis genes are typically found on chromosomes (Bruins et al., 2000; Cervantes et al., 2001).

The *mer* operon, independent of its position on the chromosome or plasmid, is a well-studied metal tolerance system that is found in diverse types of bacteria, according to Narita et al. (2003) and Reniero et al. (1998). *merA, merT, merP*, and *merR* make up the majority of the *mer* operon, while other genes like *merB, merC, merD, merE, merF*, and *merG* may also be present. According to Große et al. (2004), the *czc* operon on the PMOL30 plasmid of *R. metallidurans* CH34 encodes resistance to cadmium, zinc, and cobalt ions through the *czcCBA* genes as well as regulatory, promotor, and unknown functional genes *czcN* and *czcI*. According to Mergeay et al. (2003), *R. metallidurans* CH34 also harbors the PMOL28 mega plasmid, which encodes resistance to Co^2+^, Ni^2+^, and chromate as well as tolerance genes for Tl^+^ and Hg^2+^ and contains several previously unidentified metal resistance genes. *ArsR, ArsA, ArsD, ArsB*, and *ArsC* are just a few of the proteins that are regulated by *ars* operon and involved in arsenate tolerance, found in many different bacterial groups (Mukhopadhyay et al., 2002). Both Gram-positive (Oger et al., 2003) and Gram-negative (Lee et al., 2007) bacteria have the *cop* operon, which confers tolerance to copper ions. The *cop* operon and the cadmium tolerance *cad* system may differ in structure and location, as observed in various bacterial groups, such as *E. hirae* and *Pseudomonas* spp. (Bruins et al., 2000).

#### 4.2.6. Microbially induced carbonate precipitation

Researchers have proposed microbially induced carbonate precipitation (MICP) as a viable approach for environmentally friendly and sustainable bioremediation of metal contaminants (Dhami et al., 2013; Zhang et al., 2018). This mechanism, which involves the secretion of urease by microorganisms, offers a simple and controllable method to rapidly generate substantial amounts of carbonates. When urea is hydrolyzed by urease, CO_2_ and NH_3_ are produced, which subsequently react in the solution to produce ammonium, bicarbonate, and hydroxide. As a result, the pH increases (becomes more alkaline), and carbonate ions are formed. Under appropriate conditions of sufficient ionic activity and the presence of divalent cations, carbonate ions can precipitate out of the solution.

According to Yin et al. (2021), cadmium (Cd) and strontium (Sr), along with other metals and radionuclides, can undergo precipitation and create insoluble carbonate minerals of their own by following same pathway. Alternatively, these metals can be co-precipitated with calcium carbonate if the calcifying microorganisms are able to sustain in HM contaminated environment. Certain HM ions, such as Cu^2+^, Cd^2+^, Pb^2+^, Zn^2+^, and Sr^2+^, which have ion radii similar to that of Ca^2+^ can be integrated into the crystal lattice of CaCO_3_ through isomorphic substitution of Ca^2+^ or by penetrating crystal interstices or defects (Kim et al., 2021). As a result, the HMs transform from soluble state of ions into insoluble state, effectively prevent their release back into the environment. The wide-ranging application of MICP and its capacity to sequester HMs establish it as a feasible *in situ* remediation technique for HMs polluted sites. Additionally, its resilience to changes in redox potential in the surrounding environment contributes to its high effectiveness and long-term stability in bioremediation (Lauchnor et al., 2013).

### 4.3. Other microbial detoxification mechanism

**FIGURE 2:**
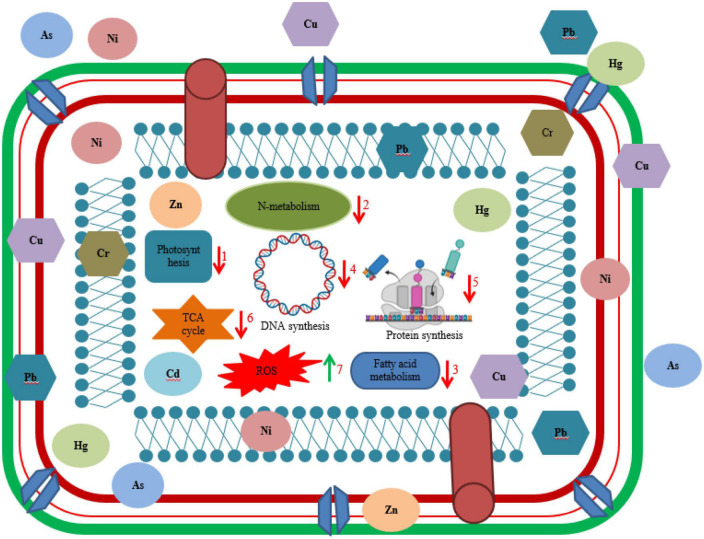
Illustration of interaction of living cell with heavy metals. Different biochemical dysfunctioning of cell occurred due to toxicity of heavy metal such as (1) decrease in rate of photosynthesis by inhibition of photosynthetic enzymes and chlorophyll biosynthesis, (2) significant decrease in nitrogen metabolism, (3) decreased rate of fatty acid metabolism, (4) disruption of DNA synthesis, (5) inhibition of protein synthesis, (6) negatively affecting key enzymes in TCA cycle, and (7) overproduction of reactive oxygen species (ROS).

Microorganisms survive via different mechanisms in the presence of HMs to resist metal toxicity as shown in Figure 2. Some commonly used strategies employed by microbes include extrusion, exopolysaccharide secretion, biotransformation, metallothionein as well as enzyme synthesis (Dixit et al., 2015). Furthermore, major mechanisms to resist HMs by microorganism involves several procedures like ion exchange, metal efflux pumps, metal oxidation, electrostatic interaction, methylation, redox process, metal-organic complexion, precipitation, exclusion by permeability barrier, metal ligand degradation, surface complexation, demethylation, metal sequestration, and bio surfactant production. Detoxification of metals by microbes can also be done by extracellular chemical precipitation, volatilization, and valence conversion (Ramasamy et al., 2006; Yang et al., 2015). Microbes have negatively charged groups on their cell surface like sulfonate, hydroxyl, alcohol, phosphoryl, carboxyl, thioether, sulfhydryl, amine, ester, and thiol implicated in metal adsorption (Gavrilescu, 2004). Few studies reported ability of bacteria to accumulate metals intracellularly such as sequestration of zinc, cadmium, and copper ions by cadmium-tolerant *E. coli*, *P. putida* strain (Hussain et al., 2022), and *R. leguminosarum* cells (Pereira et al., 2006).

Accumulation of metal ions extracellularly occurs by complexation as insoluble compounds or via cellular components in the periplasm. Several species belonging to *Geobacter* and *Desulfuromonas* are efficient to convert extremely hazardous metals to their benign forms. Some studies showing extracellular sequestration of HMs includes removal of copper ions by copper-resistant *P. syringae*, zinc ions by *Synechocystis* PCC 6803 strain, reduction of lethal forms of Manganese (IV), Uranium (VI), and Chromium (VI) to their non-toxic forms Manganese (II), Uranium (IV), and Chromium (III) by application of *Geobacter metallireducens*, a strict anaerobe (Igiri et al., 2018). Removal of cadmium (Cd) ions by *Klebsiella planticola* and *P. aeruginosa* as well as lead by the *Vibrio harveyi* strain via precipitation has been stated by several researchers (Sharma et al., 2000; Wang et al., 2002; Mire et al., 2004). Non-viable cells of *Brevibacterium* sp., *P. putida*, and *Bacillus* sp., showed effective biosorption of numerous HM ions (Green-Ruiz, 2006). Methylation by microbes play a crucial role in remediation of HMs. Methylated substances are frequently explosive; for example, *Escherichia* spp., *Pseudomonas* spp., *Clostridium* spp., and *Bacillus* spp., can bio methylate mercury (II) to gaseous methyl mercury. In polluted topsoil, bio methylation of arsenic, lead, and selenium to gaseous arsines, dimethyl lead and dimethyl selenide was observed, respectively (Ramasamy et al., 2006).

## 5. Bioremediation agents based on PGPB

Bioaccumulation and biosorptive abilities of metal-resistant bacteria are used to remediate HM-contaminated environments (Kim et al., 2015). Several properties of bacteria such as, high surface-to-volume ratios, wide distribution, capacity to thrive in controlled environments, active chemisorption sites, size, and resistance to environmental conditions contribute to their robust biosorption potentiality (Srivastava et al., 2015; Mosa et al., 2016).

Bacteria and fungi are also reported to produces organic acids as a natural chelating agent for HMs (Seneviratne et al., 2017). Gluconic, acetic, oxalic, and malic acids are the most commonly reported organic acids for HM solubilization (Ullah et al., 2015; Gube, 2016). Out of single, consortium or immobilized forms of bacterial culture consortiums are observed to be more stable, metabolically superior, survive longer for metal biosorption and suited more for field use (Gu and Berry, 1991; Kader et al., 2007). Some studies demonstrating remediation of HM using bacteria includes removal of chromium (Cr) by consortia of *Acinetobacter* sp. and *Arthrobacter* sp. (De et al., 2008), cadmium, lead, and chromium removal in tannery effluent by mixed culture of *B. megaterium*, *Penicillium* sp., *B. subtilis*, and *Aspergillus niger* (Abioye et al., 2018), Pb removal by *Micrococcus luteus* (Puyen et al., 2012), removal of Cr^6+^, Cu, and Ni utilizing zeolite immobilized *Desulfovibrio desulfuricans* (Kim et al., 2015).

Biofilms are also proven as effective bioremediating agent, biological stabilizer as well as possess high tolerance against toxic compounds at lethal quantities. Biofilms act as biosorbents or secrete exo-polymeric substances having surfactant or emulsifier properties for remediation of HMs (El-Masry et al., 2004). Several issues faced due to metallic stress includes reduced soil microbial activity as well as crop output (Ahemad, 2012), oxidative stress, modification, and loss of protein functionality leading to growth impairment, browning of roots, photosystems inactivation, and chlorosis in plants (Shaw et al., 2004; Göhre and Paszkowski, 2006; Seth et al., 2008). PGPB are well known to have adapted mechanisms for removal of metal pollutants like oxidation–reduction, biosorption, complexation, and precipitation (Muñoz et al., 2006; Singh et al., 2010). The bacterial response to a specific HM in the cleanup of metal-contaminated locations is of significant use (Hemambika et al., 2011). Various studies reported use of PGPB as bioremediating agents such as remediation of copper toxicity by *Azospirillum lipoferum* (UAP154 and UAP40), *R. leguminosarum* CPMex46 and *Pseudomonas fluorescens* Avm, in alfalfa *Medicago sativa* seeds (Carrillo-Castañeda et al., 2005); nickel toxicity by *Microbacterium arabinogalactanolyticum, Sphingomonas macrogoltabidus*, and *Microbacterium liquefaciens* in *Alyssum murale* plants (Abou-Shanab et al., 2003). Furthermore, another study showed that hydroxamate siderophores produced by PGPB strains chelates HMs present in rhizospheric region of plants leading to inhibition of free radical formation and prevention of oxidative damage to plants (Dimkpa et al., 2009). Tripathi et al. (2005) reported growth promotion as well as inhibition of lead and cadmium toxicity by *P. putida* in *Phaseolus vulgaris*. Similarly, inoculation with *Pseudomonas* sp. Ps29C and *B. megaterium* Bm4C showed plant growth promotion and low nickel toxicity in *Brassica juncea* (Rajkumar and Freitas, 2008). Barzanti et al. (2007) found that bacteria aided plant growth under nickel stress, which is consistent with previous observations. In all, these investigations clearly demonstrated the potential of PGPB to improve plant biomass under HM stress. As a result, using metal detoxifying PGPB in conjunction with other plant growth-promoting activities can significantly improve the efficiency of the entire remediation process (Glick, 2012).

Rhizoremediation involves crucial role of rhizomicrobial population in detoxification process of HM in contaminated soils (Kuiper et al., 2004). These microbes exhibit high metabolic activity around the roots of plants. Bacterial population dominating HMs stressed sites includes *Pseudomonas, Rhizobia, Arthrobacter*, and *Bacillus* (Pires et al., 2017). One of the most well-known symbiotic associations found between legume and rhizobia is capable to remediate HM toxicity and improve the condition of contaminated soils (Checcucci et al., 2017). They have the potential to transform a wide range of metals and alter metal dissolution, toxicity, speciation, mobility, and degradation in soil (Gadd, 2010). Several reviews are available on metal–microbe’s interaction by Giller et al. (2009), Khan (2005), Gadd (2010), and Kong and Glick (2017). Interaction between microorganisms and metals in the rhizosphere is highly specific and influenced by physico-chemical properties of soil, soil type, metabolic activity, concentration, type of metal species, and microbial diversity.

## 6. Conclusion

Globalization and the technological improvement is achieving its height day-by-day for the convenience to the humankind. But its use is not done at an optimal level or judicially by the generation due to which its toxic components accumulating exponentially in the environment are affecting human health as well as agroecosystems by causing toxicity. Bioremediation using indigenous microorganisms from contaminated sites seems prudent, for exploiting their true potential. Several strategies including, oxidation, reduction, condensation, hydrolysis, isomerization, chelation, precipitation, complexation, immobilization, adsorption, bioaccumulation, and production of biosurfactant are expressed by microbial system for the removal or transformation of toxic HM into their non-toxic state in the contaminated sites. Preventive measures must be taken to avoid use of metal containing pesticides in agriculture and proper monitoring must be done before releasing metal containing effluents into water bodies. For future prospects, the use of genetically modified microorganisms could remove HM efficiently from the contaminated sites. Hence need to develop some other biological based techniques which efficiently work at contaminated sites and transformation of research from laboratory level to field or industrial level is also required.

## Author contributions

All authors listed have made a substantial, direct, and intellectual contribution to the work, and approved it for publication.
